# Comparing the short - term outcomes and complications of monopolar and bipolar transurethral resection of bladder tumors in patients with coronary artery disese: a prospective, randomized, controlled study

**DOI:** 10.1590/S1677-5538.IBJU.2017.0309

**Published:** 2018

**Authors:** Deniz Bolat, Bulent Gunlusoy, Ozgu Aydogdu, Mehmet Erhan Aydin, Cetin Dincel

**Affiliations:** 1 Department of Urology, Bozyaka Training and Research Hospital, Izmir, Turkey

**Keywords:** Urinary Bladder Neoplasms, Prostatectomy, Coronary Artery Disease

## Abstract

**Introduction:**

To compare the perioperative outcomes and complications of monopolar and bipolar transurethral resection of bladder tumors (TURBT) in patients with coronary artery disease (CAD).

**Materials and Methods:**

A total of 90 CAD patients with newly diagnosed bladder cancer who underwent TURBT were randomized into monopolar TURBT (M-TURBT) and bipolar TURBT (B-TURBT) groups. Primary outcome was safety of the procedures including obturator jerk, bladder perforation, clot retention, febrile urinary tract infection and TUR syndrome. The secondary outcome was the efficacy of TURBT procedures, including complete tumor resection, sampling of the deep muscle tissue and sampling of the qualified tissues without any thermal damage.

**Results:**

Mean ages of the patients in M-TURBT and B-TURBT groups were 71.36±7.49 and 73.71±8.15 years, respectively (p=0.157). No significant differences were found between M-TURBT and B-TURBT groups regarding complete tumor resection (76.2% vs. 87.5%, p=0.162) and muscle tissue sampling rates (71.4% vs. 64.6%,p=0.252). Obturator jerk was detected in 16.7% of the patients in M-TURBT group and 2.1% in B-TURBT group (p=0.007). No statistically significant differences were found between the groups regarding intraoperative and postoperative complications.

**Conclusions:**

Both monopolar and bipolar systems can be used safely and effectively during TURBT procedure in CAD patients. Due to the more frequently seen obturator jerk in M-TURBT than B-TURBT, careful surgical approach is needed during M-TURBT.

## INTRODUCTION

Transurethral resection (TUR) is the cornerstone of diagnosis and initial therapy of the bladder tumors (1). The aim of the transurethral resection of bladder tumors (TURBT) is to reach a definitive diagnosis and to remove all visible lesions, including part of the underlying muscle tissue (2). Traditionally, TURBT has been performed with monopolar cautery. The potential hazards of this modality include hypotonic fluid absorption and the resultant electrolyte imbalance (3). Recently, the bipolar resectoscope used for TUR of the prostate (TURP) was introduced for the treatment of bladder tumors (2). Bipolar technologies allow the electric current to complete the circuit without passing through the patient (4). By this way, saline solution can be used instead of glycine for irrigation during resection. Initial studies of bipolar TURBT (B-TURBT) were promising with fewer fluid and electrolyte abnormalities, and a decreased incidence of obturator jerk (5-7).

With the progressive aging of the population, the prevalance of vascular diseases is increasing (8). In their study, Lucia et al. reported that TURP had low risk for severe complications, but cardiovascular events in elderly patients undergoing this surgical operation were more common than in general population (9). With an increase in the number of elderly population that requires surgical procedures and anticoagulant/ antiaggregant therapy, these patients need great concern for possible complications related to accompanied comorbidities (10). Patients with bladder tumors are as older as patients with benign prostatic hyperplasia (BPH). An important feature of these patients is coronary artery disease (CAD) requiring anticoagulant/antiaggregant therapy and thus having serious risk factors for possible perioperative bleeding complications. No consensus exists among urologists regarding the pre-, intra-, and postoperative management of patients taking anticoagulant/antiaggregant drugs (11). The surgical procedure could not be postponed due to the risks of progression of the malignancy and acute bleeding (10).

In this prospective, randomized, and controlled study, our aim was to compare the short-term outcomes and complications of monopolar and bipolar TURBT in patients with CAD.

## MATERIALS AND METHODS

Design: The Institutional Review Board approved this single-center, prospective, randomized and controlled study in March 2015. Written informed consent form was obtained from all patients prior to the study.

Between March-December-2015, a total of 90 consecutive patients who underwent TURBT for overt or suspected bladder cancers on radiological imagings and/or cystoscopy were enrolled in the study. All patients had grade 2 or 3 CAD according to New York Heart Association's (NYHA) classification.

Before the operation, a detailed consultation of patients were done by a cardiologist and an anesthesiologist. Anticoagulant/antiaggregant medications such as acetylsalicylic acid (ASA), warfarin, or clopidogrel were stopped before 7 days of the procedure and, if necessary, replaced by low-molecular-weight heparin.

Exclusion criteria: Patients without CAD were excluded. Also, patients with acute urinary tract infection, absence of urethelial cancer on pathology report after TURBT, who underwent TURBT for residual tumors, re-staging or recurrent bladder tumors and who were not suitable for spinal anesthesia were excluded.

Randomization: The patients were equally randomized, by means of sealed envelopes, for monopolar TURBT (M-TURBT) or bipolar TURBT (B-TURBT). Patients were blinded to the allocated group.

Technique of M-TURMT and B-TURMT: At dorsal lithotomy position, perineal skin was cleaned with the antiseptic solution. No obturator nerve block was performed before the procedure. Under spinal anesthesia, after a routine cystourethroscopy, M-TURMT was performed with an U-shaped cutting loop, 26Fr continuous flow resectoscope (Karl Storz Endoskope, Tuttlingen, Germany) with 30-degree telescope, and an electrosurgical generator (Valleylab Force FX, Boulder, CO, USA) with power settings of 120W for cutting and 80W for coagulating using mannitol irrigation. Differently, in patients who underwent B-TURBT, an ESG-400 bipolar generator (Olympus Europe, Hamburg, Germany) with power settings of 200W for cutting and 120W for coagulating with saline irrigation was used. At the end of the operation 22Fr 3-way Foley catheter was placed in all patients, and if indicated, continuous irrigation saline was maintained until the urine efflux was completely clean. In uncomplicated cases at the postoperative 24 to 48 hours, Foley catheter was removed and the patient was discharged.

Outcomes: Primary outcome of this study was the safety of the procedures including obturator jerk, bladder perforation, clot retention, febrile urinary tract infection and TUR syndrome. Severity of obturator jerk was classified based on our previous study (12). If the adductor spasm was severe enough to disturb the surgeon's resection, it was deemed as a severe obturator jerk. However, if there was an adductor spasm, but not severe enough to disturb the surgeon, it was deemed as a moderate obturator jerk. Bladder perforation was defined as subserosal injury if the perivesical fatty tissue was seen and as complete perforation if drainage tube or surgical repair was required. TUR syndrome was defined as serum sodium level <125mmol/L and one or more circulatory and/or neurological symptoms.

The secondary outcome was the efficacy of both TURBT procedures, including complete tumor resection, sampling of the deep muscle tissue and sampling qualified tissues without any thermal damage. All resections were performed under the supervision of a senior urologist and resection completeness and complications were noted intraoperatively. Thermal damage was classified into 2 groups depending on the quantity of cautery artifacts: mild cautery artifact was defined as cautery artifacts involving less than 50% of entire specimen, and severe cautery artifact was defined as cautery artifacts involving more than 50% of entire specimen (1).

Pathological examination: A single uropathologist, blinded for the allocation, evaluated the resected specimens. Tumor stage, tumor grade, presence of muscularis propria, invasion of the muscle tissue, and presence of thermal tissue damage were reported (13, 14).

Statistical analysis: Data was analysed using the Statistical Package for Social Sciences (SPSS 17.0 for Windows, Chicago, IL, USA). Power calculations were performed with minitab 17 software. Data was expressed as mean±standard deviation, number and percentage according to the type of variables. Numeric variables were tested using independent sample T test, and categorical variables were tested using chi-square or Fisher's exact test. Values of p <0.05 were accepted as statistically significant.

## RESULTS

**Figure 1:**
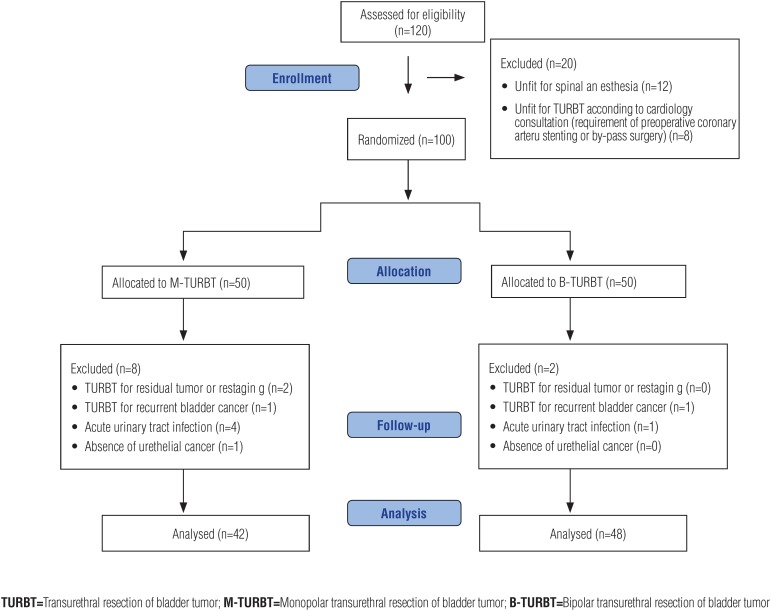
CONSORT (Consolidated Standards for Reporting Trials) flow diagram for patient assessment, allocation, follow-up and analysis.

A total of 120 patients was enrolled in the study. Twenty patients were excluded from the study prior to the randomization. Of the excluded patients, 12 of them were unfit for spinal anesthesia and 8 of them were unfit for TURBT operation due to the priority requirement of coronary artery stenting or by-pass surgery. A total of 100 patients were equally randomized to M-TURBT and B-TURBT groups. After randomization, 10 patients were excluded bacause of active urinary tract infection, absence of urethelial cancer, and TURBT for residual or recurrent bladder cancer. Finally, 42 patients in M-TURBT group and 48 patients in B-TURBT group were analyzed (Figure-1).

Of the patients, 42 in M-TURBT group and 48 in B-TURBT group had only CAD or CAD and concomitant diseases, such as hypertension (HT) and/or diabetes mellitus (DM). No statistically significant differences were observed in the baseline characteristics of 2 groups. Mean ages in M-TURBT and B-TURBT groups were 71.36±7.49 and 73.71±8.15 years, respectively (p=0.157). Mean tumor sizes were 3.1±2.4cm in M-TURBT group and 3.0±2.9cm in B-TURBT group (p=0.875). Mean tumor numbers were 1.8±1.4 and 2.0±1.6 in M-TURBT and B-TURBT groups, respectively (p=0.556). Patient's characteristics are shown in Table-1.

**Table 1:** Preoperative patient characteristics.

		M-TURBT	B-TURBT	P
**No. patients**		**42**	**48**	**0.173** *
	Male	37 (88.1)	37 (77.1)	
	Female	5 (11.9)	11 (22.9)	
Mean±SD age (years)		71.36±7.49	73.71±8.15	0.157+
Mean±SD BMI (kg/m^2^)		25.14±4.80	26.13±4.82	0.336+
Comorbidities		42	48	0.173*
Only CAD		18	20	
CAD+HT		16	21	
CAD+DM		5	4	
CAD+HT+DM		3	3	
**No. NYHA score**				**0.955** *
	2	19 (45.2)	22 (45.8)	
	3	23 (54.8)	26 (54.2)	
**No. ASA score**				**0.426** *
	1	0	0	
	2	21(50)	19 (39.6)	
	3	21(50)	28 (58.3)	
	4	0	1 (2.1)	
Mean±SD preop Hb (g/dL)		13.3±1.8	13.0±1.9	0.143†
Mean±SD preop Na (mmol/L)		137.6±2.2	137.8±3.1	0.736†
Mean±SD tumor size (cm)		3.1±2.4	3.0±2.9	0.875†
Mean±SD tumor number		1.8±1.4	2.0±1.6	0.556†
No. orifice involvement		10 (23.8)	14 (29.2)	0.566*
**No. stage**				**0.473** *
	Ta	24	22	
	T1	12	15	
	T2	6	11	
	Tis	1	1	
**No. grade**				**0.530** *
PUNLMP		8	11	
Low		23	19	
High		11	18	

**BMI**=Body mass index; **CAD**=Coronary artery disease; **HT**=Hypertension; **DM**=Diabetes Mellitus; **ASA**=American Society of Anesthesiologists; **NYHA**=New York Heart Association; **Hb**=Hemoglobin; **Na**=Sodium

† Independent sample t test

* Chi-square

The operation time was not significantly different between M-TURBT and B-TURBT groups (34.6±18.7min vs. 34.3±21.2min; p=0.955). Obturator jerk was detected in 16.7% of the patients in group 1 and 2.1% in group 2, and this difference was statistically significant (p=0.007). No significant differences were found between M-TURBT and B-TURBT groups regarding complete tumor resection rates (76.2% vs. 87.5%, p=0.162) and muscle tissue sampling rates (71.4% vs. 64.6%,p=0.252). There were only two patients with thermal tissue damage in M-TURMT group and 1 patient in B-TURBT group. Mean cateterization time (days) was 1.7±1.4 and 1.5±1.3 in M-TURBT and B-TURBT groups, respectively (0.948). Intraoperative and postoperative outcomes are displayed in Table-2.

**Table 2:** Intraoperative and postoperative outcomes.

		M-TURBT	B-TURBT	P
Mean±SD operation time (min.)		34.6±18.7	34.3±21.2	0.955†
Mean±SD resected tissue (cc)		3.77±4.47	5.55±9.52	0.251†
**Obturator jerk (%)**				0.007*
	Moderate	5 (11.9)	1 (2.1)	
	Severe	2 (4.8)	0	
No. complete tumor resection		32 (76.2)	42 (87.5)	0.162*
No. early postop instillation		14 (33.3)	8 (16.7)	0.074**
**No. thermal tissue damage (%)**				1.000*
	Grade 1	1(2.4)	1 (2.1)	
	Grade 2	1 (2.4)	0	
No. muscle tissue sampling (%)		30 (71.4)	31(64.6)	0.252*
Mean±SD cateterization time (day)		1.7±1.4	1.5±1.3	0.948†
Mean±SD hospitalization time (day)		2.8±2.1	2.4±2.1	0.456†

† Independent sample t test;

* Chi-square;

** Fisher's exact

Subserosal bladder injury was detected in 6 patients (14.3%) in M-TURBT and 4 patients (8.3%) in B-TURBT groups (p=0.505). Complete bladder perforation was not detected in any of the groups. No significant differences were observed between the groups regarding clot retention, requirement of blood transfusion and recoagulation. There was no patient with TUR syndrome in each group. Mean hemoglobin (Hb) and sodium (Na) decreases in the postoperative period were comparable between the groups. Postoperative complications and adverse events are displayed in Table-3.

**Table 3:** Postoperative complications and adverse events.

	M-TURBT	B-TURBT	P
No. bladder perforation (%)			0.505*
Subserosal injury	6 (14.3)	4 (8.3)	
Complete perforation	0	0	
No. febrile urinary tract infection (%)	0	0	
No. clot retention (%)	1 (2.4)	0	0.461**
No. blood transfusion (%)	2 (4.8)	0	0.215**
No. TUR syndrome (%)	0	0	
No. recoagulation (%)	1 (2.4)	0	0.461**
Mean ± SD Hb decrease (g/dL)	-0.66±0.66	-0.72±1.24	0.307†
Mean ± SD Na decrease (mmol/L)	-0.40±2.64	-0.43±3.40	0.051 †

**TUR=**Transurethral resection, Hb=Hemoglobin), Na=Sodium

† Independent sample t test;

* Chi-square;

** Fisher's exact

In the peri-and postoperative periods, there was no death in both groups.

## DISCUSSION

Uncomplicated surgery is especially important in urology clinics, which assist an elderly patient population. Even a small complication that develops in this patient group can lead to serious consequences due to accompanying comorbidities. The most common deadly comorbidity in the elderly patient group is CAD with or without comorbidities such as HT and DM. Overall, urological surgery is associated with a 2% risk of postoperative myocardial infarction and cardiac-related mortality, with TURBT considered as a low risk operation (15). In this comparative study, we aimed to analyze the results of monopolar and bipolar TURBT in patients who had CAD with or without other comorbidities.

Transurethral resection is integral to the management of bladder neoplasms (1). In recent years, as their experience with bipolar resection systems increases, urologists have begun to prefer these systems in the treatment of bladder tumors. A bipolar current has better hemostatic capacity compared with a monopolar current because it allows deep coagulation and has a cut and seal effect (16). Sugihara et al. found a similar incidence of postoperative hemostatic procedures and transfusion rates in a comparative study between monopolar and bipolar TURBT (16). In another study, Zhao et al. reported that bleeding was not a severe risk factor during TURBT to the urologists in the current approaches, and the established blood loss between both groups was not significantly different (17). Based on the prostatic experience, bipolar resection was believed to induce better hemostasis as the deep coagulation and the cut and seal property of the bipolar current contribute to augment the ability to control bleeding points (18, 19). In the current study, mean Hb and sodium decreases in the postoperative period were not different between the two groups. This can be explained by three facts: Firstly, bleeding in TUR-BT was not severe as that in TURP, secondly the amount of tissue resected in bladder tumors was much less than in prostate operations, and lastly the operation time of TURBT is shorter than TURP. When we examined postoperative complications, blood transfusion was performed in 2 patients and clot retention was seen in one patient in M-TURBT group. None of the patients had blood transfusion and clot retention in B-TURBT group. In our series, the mean catheterization time and hospitalization time were also similar in both groups.

A major concern for most urologists is to achieve complete removal of the bladder tumor without any complications (20). An incidence of obturator jerk during TURBT is variable according to the type of anesthesia employed and the site of the tumor (21). Reporting the incidence and the difference between both techniques in inducing obturator reflex is the subject of debate (21). Some reports described obturator reflex occured in nearly half of the patients and others reported an incidence around 1% (2, 22). Aggresive, deep resection or obturator nerve reflex that results in violent adduction of the leg during the resection may cause the injury or even perforation of the bladder wall (23). In the current study, the incidence of obturator reflex was significantly different in each group and found as 16.7% in group 1 and 2.1% in group 2 (p=0.007). Spinal anesthesia without obturator block carries higher risk than general anesthesia for obturator jerk. Although it is rare, bladder perforation is a major complication that worries urologists. Real incidence of bladder perforation might be possibly underestimated because of underreporting, and it ranges from 1.7 to 5% (6, 24). Similar to obturator reflex debate, the value of bipolar resection in decreasing the incidence of bladder perforation is yet to be confirmed (21). Gupta et al. reported a significant rate of obturator jerks and subsequent perforation in their first 10 patients when the power setting of the bipolar machine was adjusted for 160 and 80W for cutting and coagulation, respectively (25). But, they showed that such complications had been eliminated by using a lower power setting of 50 and 40W (25). In another study, Golan et al. reported an incidence of 0.36% for bladder perforation in an analysis of 4.144 TURBT procedures and concluded that severe bladder injury was more likely to occur in elderly patients with large tumors located on the posterior wall, and that it did not appear to increase the risk of extravesical seeding (26). In this study, none of the patients in both groups had complete perforation of bladder.

None of our patients died from cardiovascular disease. Undoubtedly, this can not be solely attributed to the success of the surgical procedure. It is important to evaluate these patients carefully and rigorously for thelast cardiac status in the preoperative period. The operation may be delayed temporarily in patients who require urgent coronary stenting or have severe arrhythmia, except those who need immediate surgical intervention. Preoperative withdrawl of anticoagulant/antiaggregant drugs to reduce the risk of regional or neuraxial blockade is another important point. The interruption of long-term acetyl salicylic acid (ASA) treatment for elective urologic procedures creates a management dilemma due to the competing risks of recurrent ischemic events and hemorrhage (10). In a meta-analysis of 50.279 patients treated with ASA for secondary prevention of CAD, Eisenstein et al. showed that the cardiac complication rate increased threefold after withdrawl of ASA and that the rate was even higher for patients with coronary stents (27). Despite being controversial, our clinical approach is to stop anticoagulant/antiaggregant medicines before 7 days and give low-molecular-weight heparin for prophylaxis. Our results support that this clinical approach is feasible for these group of patients. To the best of our knowledge, the recent study represents the first trial comparing the safety and efficacy of monopolar and bipolar TURBT in patients with CAD.

Our study has some limitations. Firstly, the present study has relatively small sample size. The low number of the patients can be explained by our exclusion criteria. We excluded patients who were not suitable for spinal anesthesia and unfit for surgery due to previous coronary artery stenting or by-pass surgery. We also excluded patients who were treated with anticoagulant/antiaggregant medication. Since most of the patients with CAD had to use anticoagulant/antiaggregant drugs, our final patient number was 90. Secondly, we had supposed that anticoagulant/antiaggregant medication could potentially effect the outcomes of this prospective study and excluded these patients to have a more homogenous group of patients.

## CONCLUSIONS

Both monopolar and bipolar systems can be used safely and effectively during TURBT procedure in patients with CAD. Obturator jerk was more frequently seen in M-TURBT and a careful surgical approach is needed during M-TURBT.
